# Peroxymonosulphate Activation by Basolite^®^ F-300 for *Escherichia coli* Disinfection and Antipyrine Degradation

**DOI:** 10.3390/ijerph19116852

**Published:** 2022-06-03

**Authors:** Antía Fdez-Sanromán, Marta Pazos, Angeles Sanroman

**Affiliations:** CINTECX, Department of Chemical Engineering, Campus As Lagoas-Marcosende, Universidade de Vigo, 36310 Vigo, Spain; antia.fernandez.sanroman@uvigo.es (A.F.-S.); mcurras@uvigo.es (M.P.)

**Keywords:** Advanced Oxidation Processes, antipyrine, Basolite^®^ F-300, *Escherichia coli*, MOF, disinfection, sulphate radicals

## Abstract

In this study, the removal of persistent emerging and dangerous pollutants (pharmaceuticals and pathogens) in synthetic wastewater was evaluated by the application of heterogeneous Advanced Oxidation Processes. To do that, a Metal-Organic Framework (MOF), Basolite^®^ F-300 was selected as a catalyst and combined with peroxymonosulfate (PMS) as oxidants in order to generate sulphate radicals. Several key parameters such as the PMS and Basolite^®^ F-300 concentration were evaluated and optimized using a Central Composite Experimental Design for response surface methodology for the inactivation of *Escherichia coli*. The assessment of the degradation of an analgesic and antipyretic pharmaceutical, antipyrine, revealed that is necessary to increase the concentration of PMS and amount of Basolite^®^ F-300, in order to diminish the treatment time. Finally, the PMS-Basolite^®^ F-300 system can be used for at least four cycles without a reduction in its ability to disinfect and degrade persistent emerging and dangerous pollutants such as pharmaceuticals and pathogens.

## 1. Introduction

Currently, as a consequence of anthropogenic activities, many active pharmaceutical ingredients are present in various aqueous matrix waters such as wastewater (from hospitals and urban areas), surface water, and groundwater [1]. Although they have been spotted at low concentrations, with the range from ng to μg, there are indications that they may be toxic to living organisms [2,3,4]. Moreover, to complicate this environmental situation, the removal efficiency of pharmaceuticals in Waste Water Treatment Plants (WWTP) is variable and some of these compounds pass through these plants almost intact [5,6]. Indeed, this inefficiency in the elimination of pharmaceuticals has also been proven to occur with pathogens, such as SARS-CoV-2, that have been detected in WWTPs [7,8] and used as an early warning indicator for the COVID-19 pandemic [9]. The increase of water pollution by infectious biological contaminants is a serious health risk around the globe. The pathogens may enter wastewater systems from different via such as human waste, the introduction of decontamination wastewater, illicit activity, animal farming, and hospital effluents, or surface water runoff following a wide-area biological incident [10,11,12,13]. Thus, hospitals are considered a source of various pathogens [14] and pharmaceuticals [15], as well as radioactive elements or toxic compounds [16]. In addition, recent studies have detected antibiotic resistance genes (ARG) and bacteria (ARB) in hospital wastewater [17]. Conventional disinfection methods such as chlorination and ultraviolet light radiation effectively eliminate some ARG and ARB, but not when there is a high concentration of bacteria in wastewaters [18,19]. In this case, these resistant bacteria would be in a state where they are able to live but not grow. The first bacteria found in these states, so-called viable but non-culturable states, were *Escherichia coli (E. coli)* and *Vibrio cholera* [19].

Another current problem is the overuse of water, which may lead to a lack of it as a resource [20]. Thus, it is crucial to improve the quality of treated wastewater to enable reuse in industries and agriculture [21,22,23], being essential to reduce the negative impact of pharmaceuticals and pathogens in the aquatic environment [24].

Among the current alternatives to the conventional technologies, Advanced Oxidation Processes (AOPs) are highlighted due to their eco-friendliness and high ability to disinfect and degrade persistent emerging and dangerous pollutants [25,26,27]. The AOPs use non-selective radicals of high oxidizing power. These radicals, such as hydroxyl (HO^•^) and sulphate (SO_4_^•−^) radicals, oxidize organic pollutants until their mineralization (CO_2_, H_2_O, and inorganic compounds) [28,29] and cause membrane rupture in microorganisms [30,31].

Furthermore, these AOPs can be classified according to activation methods such as heat, electrochemical [32,33], UV-Vis irradiation [34], ultrasonic [35], and transitions metals (such as Fe^2+^, Cu^2+^, Co^2+^, Ag^+^, …) [36]. Additionally, there must also be a distinction between homogeneous [37] and heterogeneous [38] systems. The use of peroxymonosulfate (PMS) stands out among these AOPs capable of generating such radicals, especially in the presence of a transition metal, as demonstrated in equation 1 [39]. It is highlighted that SO_4_^•−^ has a long lifetime (30–40 μs (SO_4_^•−^) vs. < 1 μs (HO^•^) and high redox potential (2.5-3.1 V (SO_4_^•−^) vs. 1.8–2.7 V (HO^•^)) [40,41] in a pH range between 2 and 9 [42].

(1) $$M^{n+}+{\mathrm{HSO}}_{5}^{-}\to M^{{\left(n+1\right)}^{+}}+{\mathrm{SO}}_{4}^{•-}+{\mathrm{OH}}^{-}\;$$

To improve this technology, the use of a heterogeneous catalyst is recommended due to the fact that it is easier to recover and reduce the catalysts consumption. Several heterogeneous catalysts such as clays [43], biochar [44], or hydrochar [45] have tested in AOPs. In this context, recent studies reported the properties of MOF as novel categories of porous and well-crystallined material constructed from metal cations (or clusters of metal cations) which are linked to one another by bridging organic linkers [46]. MOFs show a balanced mix of crystallinity, porosity, and tunability, and thus they have the potential to be one of the most attractive materials currently under investigation in heterogeneous catalysis [46].

In this study, the remediation of water contaminated with pathogens and pharmaceuticals will be evaluated by PMS activation using as the catalyst a commercial Fe-MOF, called Basolite^®^ F-300, as pharmaceutical products and pathogens were selected for antipyrine and *E. coli*, respectively. In this context, the Basolite^®^ F-300 amount and PMS concentration will be evaluated and optimized to improve disinfection and degradation processes.

## 2. Materials and Methods

### 2.1. Microorganism and Chemiclas

The strain used was *E. coli* CECT 102 provided by the Spanish Type Culture Collection. Basolite^®^ F-300 (C_9_H_3_FeO_6_) is produced by BASF (Ludwigshafen am Rhein, Germany), antipyrine, and PMS (2KHSO_5_·KHSO_4_·K_2_SO_4_), supplied by Sigma Aldrich (Madrid, Spain). A meat peptone broth (MPB) culture medium, which was used for both the liquid and solid medium, has the following composition: peptone, 10 g/L; meat extract, 5 g/L; and sodium chloride, 5 g/L. These mentioned chemicals were provided by Sigma Aldrich. Moreover, it also employed Eosin Methylene Blue Agar (EMB) plates, which were given by Scharlau Microbiology (Barcelona, Spain), in order to ensure that only *E. coli* was growing in the disinfection experiments described below (Section 2.3.1).

### 2.2. Culture Conditions

*E. coli* CECT 102 inoculum was transferred into a 250 mL Erlenmeyer flask, in which 50 mL of MPB medium was incubated for 20 h at 180 rpm, in 37 °C, in the dark. This culture was used as an inoculum (0.5 *v*/*v* inoculum) for the different disinfection experiments. The culture was incubated until the stationary phase (approx. 20 h) and then centrifuged at 8000× *g* rpm for 15 min (Sigma Laboratory Centrifuges, 3K18, Osterode am Harz, Alemania). This procedure assures a minimum concentration of 10^10^ colony-forming units (CFU) per mL. Subsequently, it was resuspended in 5 mL of sterile saline solution 0.9% *w*/*w*. All materials and pre-prepared solutions for the *E. coli* experiments have been sterilized in an autoclave Presoclave II (J.P. Selecta^®^, Barcelona, Spain). The working conditions are 121 °C temperature, 1 bar pressure, and the duration of each cycle is 20 min.

### 2.3. Experimental Set-Up

#### 2.3.1. Disinfection

**Figure 1:**
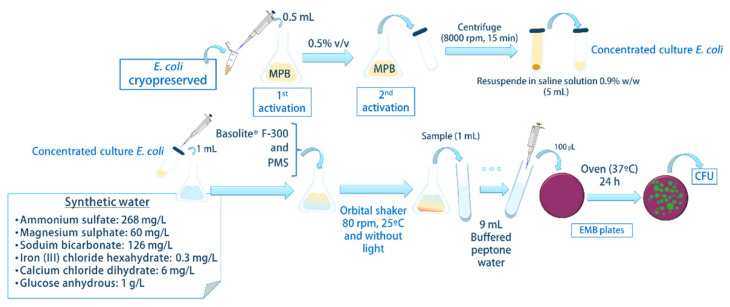
Scheme of the experimental procedure of *E. coli* disinfection.

Figure 1 shows all the steps of the disinfection experimental from the initial stages of culture growth until the final analysis to determine the efficacy of the disinfection. Two activation steps were performed. In the first, 0.5 mL of *E. coli* CECT 102 inoculum was transferred into a 250 mL Erlenmeyer flask, in which 50 mL of MPB medium was incubated for 20 h at 180 rpm, in 37 °C, in the dark, and then used as an inoculum (0.5 *v*/*v* inoculum) in the second activation step. This culture was incubated until the stationary phase (approx. 20 h) and centrifuged at 8000× *g* rpm for 15 min (Sigma Laboratory Centrifuges, 3K18, Osterode am Harz, Alemania) to obtain a concentrated culture of *E. coli*. After that, *E. coli* CECT 102 inactivation experiments were carried out at different amounts of Basolite^®^ F-300 and PMS concentrations. To do that, 1 mL of concentrated culture of *E. coli* was added to 99 mL of synthetic water and a mixture with Basolite^®^ F-300 and PMS. This mixture was kept in an incubator, MaxQ 8000 (Thermo Fisher Scientific, Madrid, Spain), at 80 rpm, in 25 °C, and in the dark for a determined time, and there was 5 min for the duration of the experiment. After these times, the bacteria concentrations were assessed by the standard plate counting method through a serial 10-fold-dilution.

#### 2.3.2. Antipyrine Degradation

The pollutant degradation experiments were carried out at different amounts of Basolite^®^ F-300 (0–263 mg/L) and PMS (0–307.4 mg/L). All experiments were carried out in 150 mL Erlenmeyer flasks with 50 mL of the pollutant solution (50 or 10 mg/L) at a natural pH and room temperature. All assays were performed in triplicate, and the reported results are the average values. Prior to degradation experiments, the adsorption of antipyrine on Basolite^®^ F-300 was evaluated. All experiments were conducted in duplicate, and liquid samples were taken out at predetermined time intervals determining the antipyrine concentration in triplicate. The average values, with a standard deviation below 5%, were considered for this study.

#### 2.3.3. Disinfection and Antipyrine Degradation Assays

A total of 100 mL of synthetic water (Figure 1) was polluted by antipyrine (10 mg/L) and *E. coli* (10^10^ CFU/mL). The efficiency of the optimized PMS-Basolite^®^ F-300 system was evaluated along the time by determination of *E. coli* and antipyrine concentration by the methods described in Section 2.4. To determine the reusability of the PMS-Basolite^®^ F-300 system, after each cycle, a concentrated solution of antipyrine and *E. coli* was added in order to start the process at the same initial concentration. In this case, the removal of *E. coli* and antipyrine were defined as the ratio between the initial and the final concentrations in each cycle.

### 2.4. Analysis

#### 2.4.1. Disinfection Efficiency

It was measured by logarithmic reduction of the survival of microorganisms (−log (N/N_0_)), where N_0_ is the concentration of viable *E. coli* obtained in the control cultures and N at the condition tested in the experiments. These concentrations of bacteria were assessed with the standard plate counting method through a serial 10-fold-dilution. All the dilutions were performed in buffered peptone water (15 g/L). Aliquots for each dilution were spread onto an EMB plate and incubated for 24 h in an oven (Memmert, UN 160, Schwabach, Germany) at 37 °C. After that, colonies were counted. Mean count values (of triplicated samples) in CFU/mL were obtained and represented, always with a coefficient of variation less than 15%.

#### 2.4.2. Antipyrine Concentration

Antipyrine decay was measured using High Performance Liquid Chromatography, HPLC (Agilent 1100, Santa Clara, CA, USA) with a Diode Array Detector at a wavelength of 242 nm. Chromatographic separation was carried out using a Kinetex^®^ column (150 mm × 4.6 mm, 5 µm Biphenyl 100 Å, Torrance, CA, USA) at room temperature. The mobile phase was a mixture of ultrapure water (65% *v*/*v*), methanol (30% *v*/*v*), and ammonium formate (100 mM) (5% *v*/*v*), operating at a flow of 1 mL/min. The injection volume was 10 µL. Before chromatographic analysis, all samples were filtered through 0.22 μm PVDF filters.

#### 2.4.3. Total Organic Carbon (TOC) Analysis

At the finality of the experiments, the evaluation of the antipyrine mineralization was made by determination of the initial and final TOC concentration. TOC was measured via catalytic high-temperature combustion by a multi N/C 3100 Autoanalyzer (Analytik Jena, Jena, Germany) coupled with a nondispersive infrared (NDIR) detector (C.A.C.T.I., University of Vigo, Vigo, Spain). Before injection, samples were acidified with H_2_SO_4_ in order to reach a pH value of 2. This fact is important to avoid inorganic carbon (carbonates and bicarbonates) interferences. From these results, the TOC removal percentage was determined according to the following Equation (2).

(2) $$TOC_{removal}\left(\%\right)=\frac{TOC_{0}-TOC_{t}}{TOC_{0}}\cdot 100$$

where *TOC*_0_ is the initial value before treatment (mg/L) and *TOC_t_* is the *TOC* at the final treatment time (mg/L).

#### 2.4.4. Microscopy Basolite^®^ F-300 Characterization

Scanning Electron Microscopy and Energy Dispersive Spectrometry (SEM/EDS) was performed on a JEOL JSM6010LA equipped with an EDS Oxford AZtecOne SEM (C.A.C.T.I., University of Vigo, Vigo, Spain).

#### 2.4.5. Iron Leaching

Iron concentration in the solution was determined by an Inductively Coupled Plasma—Optical Emission Spectrometer (ICP-OES), Perkin Elmer Optima 4300DV (C.A.C.T.I., University of Vigo, Vigo, Spain).

### 2.5. Optimization of Disinfection Process

To optimize the process, a Central Composite Experimental Design (CCD) for response surface methodology (RSM) was used. Design Expert^®^ 8.0.0 software (Stat-Ease Inc., Minneapolis, MN, USA) was used for this design. In this case, a 2-level and 2-factor design (Table 1) with three replicated at the center points conducted a total of 11 runs (Table 2). Experimental data were fitted to quadratic model Equation (3):

(3) $$y={\;b}_{0}+\sum {\mathrm{bx}}_{i}+\sum b_{\mathrm{ii}}x_{i}^{2}+\sum b_{\mathrm{ij}}x_{i}x_{j}$$

where y is the response used as a dependent variable (disinfection efficiency); x_i_ and x_j_ are the independent variables (concentration of PMS and Basolite^®^ F-300 showed in Table 1); b_0_ is the constant coefficient; b_i_ is the slope or linear effect of the input factor; b_ii_ is the quadratic effect; and b_ij_ is the two-way linear by linear interaction effect. Design Expert^®^ 8.0.0 software was also used to assess the analysis of variance (ANOVA).

## 3. Results

### 3.1. E. coli Disinfection

In this study, a CCD was adopted, since the response (disinfection efficiency) can be simply related to the key parameters such as the PMS and Basolite^®^ F-300 amount using the quadratic models shown in equation 2. The levels of PMS (x_1_) and Basolite^®^ F-300 (x_2_) were the independent variables and their values are shown in Table 2. Furthermore, in this design, three replicates of the central point have been selected to ensure this fundamental point in the design, and this experimental design was set to be face-centered with an α = 1, which means that the axial points are located on the faces of the cube. The low, center, and high levels correspond to the coded values designated as −1, 0 and 1, respectively (Table 1). The result of these specifications of this design states that it is necessary to carry out 11 runs (Table 2).

By analyzing the results of each condition after 5 min (Table 2), it is evident that the disinfection results show an upward trend for each PMS amount (30.7–153.7 mg/L) due to the addition of Basolite^®^ F-300. When PMS is 153.7 mg/L, the effect of the amount of this catalyst is easier to observe. Thus, the disinfection efficiency of the first run without Basolite^®^ F-300 is 5.096, which rises to 6.236 when the amount of Basolite^®^ F-300 present in the solution is increased to 65.8 mg/L (run 8). However, it is observed that the amount of PMS is the main and determining factor in the elimination of *E. coli* when comparing run 9 with run 1.

The regression model, as well as the analysis of variance (ANOVA), was assessed using Design Expert^®^ 8.0.0 software (Table 3). The ANOVA analysis is essential to evaluate the significance and adequacy of the model. It subdivides the total variation of the results into two sources of variation, the model and the experimental error, which show whether the variation from the model is significant when compared to the variation due to residual error.

The significance of the model and the importance of each coefficient were determined by the values of *F* and *p*-values. Moreover, these two values allow to study whether the main and interaction effects between the independent variables are statistically significant or not [47]. In this model, the confidence interval is 95%, the *F*-value is 105.45 and the *p*-value is <0.0001 (Table 3). Therefore, because the *p*-value obtained is less than 0.05 and the *F*-value is high, it is said that the model obtained made a good approximation to predict the outcome variable and there is a significant relationship between the set of predictors and the dependent variable indicating that the model has significance [48,49]. Furthermore, there is only a 0.01% chance that an *F*-value this large could occur due to noise.

Another aspect that is relevant to point out is the *p*-value used to determine the significance of each term. According to these values, the terms PMS (x_1_), Basolite^®^ F-300 (x_2_), and the square of PMS (x_1_^2^) are significant model terms [50]. The high correlation coefficient (R^2^ = 0.986) demonstrated an outstanding agreement between the predicted and experimental values. In addition, the predicted correlation coefficient (Pred R^2^ = 0.934) is in reasonable agreement with the adjusted correlation coefficient (Adj R^2^ = 0.977) [50]. It also is noted that the values of lack of fit were not significant, which reinforces that the quadratic model was adequate and fits the experimental results correctly [51].

As for the signal-to-noise ratio, this was measured by the Adeq Precision. A ratio greater than 4 is desirable and in this model, a ratio of 29.16 was obtained indicating an adequate signal. Therefore, this model can be used to navigate the design space. In addition, the percentage of the coefficient of variation (CV) was less than 10%, thus demonstrating the high accuracy and reliability of the experiments performed [52].

The obtained expression of the selected response and disinfection efficiency (after 5 min), as a function of the studied variables coded factors, is described in the Equation (4):

y = 4.86 + 1.68x_1_ + 0.37x_2_ + 0.027x_1_·x_2_ − 0.75x_1_^2^ (4)

**Figure 2:**
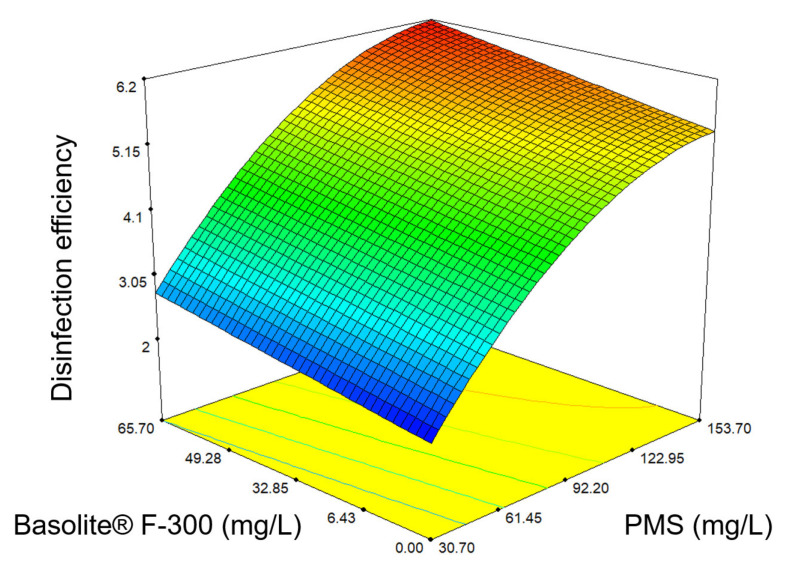
The 3D surface plot in which the response is the *E. coli* disinfection efficiency (−log(N/N_0_)) after 5 min, in the function of the PMS and Basolite^®^ F-300 concentration.

Response surface plots (Figure 2) provide a valuable tool to predict the disinfection efficiency for different values of the assessed variables and help to identify the type of interactions among these variables. Figure 2 depicted the graphical representation of the response surface of PMS and Basolite^®^ F-300 concentration. As it can be seen, the disinfection improves when the PMS and Basolite^®^ F-300 concentration increase, but the maximum is achieved when the concentration of PMS and Basolite^®^ F-300 were 153.7 mg/L and 65.8 mg/L, respectively.

### 3.2. Antipyrine Removal

#### 3.2.1. Antipyrine Adsorption

In recent decades, MOFs have gained increasing interest as novel functional inorganic–organic hybrid materials due to their high-tunability structures with significant great surface areas, facile control of porosity and cavities, and functional properties [53,54]. Thus, these properties open the possibility to use them as adsorbents for the removal of pollutants from the wastewater as has been reported by several authors [55,56]. Based on this, it is necessary to determine the ability of Basolite^®^ F-300 to adsorb antipyrine before starting the experimental trials with PMS.

**Figure 3:**
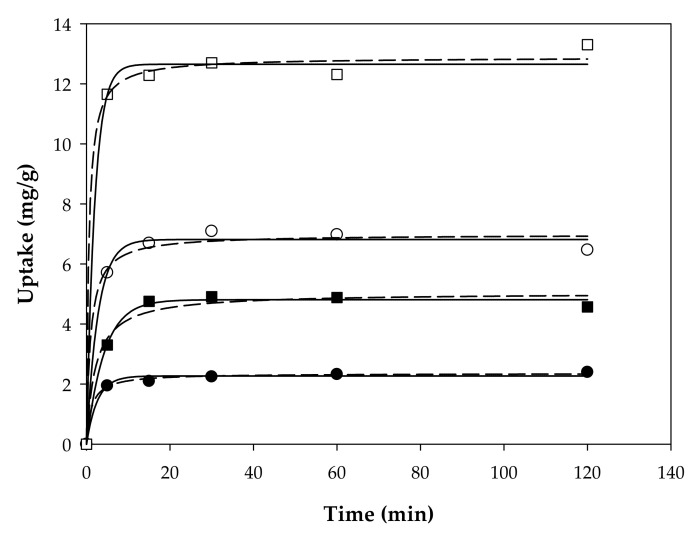
Representation of the adsorption kinetics for two concentrations of Basolite^®^ F-300 (circle: 65.8 mg/L and square: 263 mg/L), and it distinguishes the two concentrations of the pollutant between black (10 mg/L) and white (50 mg/L). Data represent the mean values for triplicate samples with a standard deviation below 5%.

Figure 3 depicted the adsorption kinetic of Basolite^®^ F-300 at two concentrations (65.8 and 263 mg/L) in a solution of antipyrine at concentrations of 10 and 50 mg/L. Two kinetic models of pseudo-first-order and pseudo-second-order kinetic models were evaluated to assess the adsorption rate, which are the following equations (Equations (5) and (6)):

(5) $$\mathrm{Pseudo}-\mathrm{first}-\mathrm{order}\;\mathrm{model}:\;q=q_{e}\cdot \left(1-e^{\left(-k_{1}\cdot t\right)}\right)$$

(6) $$\mathrm{Pseudo}-\mathrm{second}-\mathrm{order}\;\mathrm{model}:\;q=\frac{t}{\frac{1}{q_{e}^{2}\cdot k_{2}}+\frac{t}{q_{e}}}$$

where *q* and *q_e_* are the uptakes defined as amount of antipyrine adsorbed onto Basolite^®^ F-300 (mg/g) at time t and in the equilibrium, respectively; *k*_1_ (min^−1^) and *k*_2_ (g/mg min) are the pseudo-first-order rate constant and the pseudo-second-order rate constant, respectively.

The kinetics parameters from the fitting curves are listed in Table 4. In all cases, the adsorption capacities increased rapidly during the first 10 min and reached adsorption equilibrium after 30 min. The results showed that the pseudo-first-order model matches better with the experimental data with a good correlation coefficient (the average of the model selected is 0.994). In addition, the calculated *q_e_* values are in good agreement with the experimental values. Nevertheless, these results highlighted the low adsorption capacity of Basolite^®^ F-300 for the removal of antipyrine with maximum removal of 7%.

Some studies tried the removal of antipyrine by adsorption and achieved better percentages. However, it is of interest that they used different adsorbents of the Basolite^®^ F-300. For example, the capacity of adsorption of FeCl_3_-activation of Tara gum was around 275 mg/g [57], and the NQ60 aerogel, that is, a monolithic carbonaceous aerogel, was 49.7 mg/g [58]. Another adsorbent that presents better adsorption uptake of antipyrine was the optimized hydrochar from loquat cores that achieved a maximum removal of 76% [59].

Basolite^®^ F-300 has not been reported in the adsorption of pharmaceuticals, however, it is an interesting option for the removal of some metals from wastewater, such as arsenate (V), which reaches an uptake of 169.2 mg/g [60]. Nevertheless, it should be noted that there are not many scientific articles on wastewater treatment in which this MOF was used.

#### 3.2.2. Antipyrine Degradation in PMS-Basolite^®^ F-300 System

**Figure 4:**
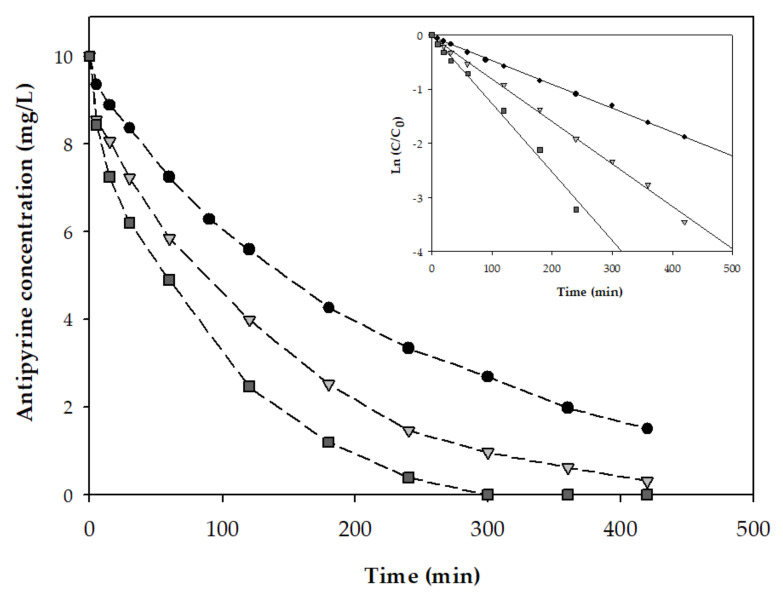
Antipyrine’s degradation profiles for three different concentrations of PMS (circle: 153.7 mg/L, triangle: 230.5 mg/L, and square: 307.4 mg/L). All experiments were made with the same concentration of Basolite^®^ F-300 (65.8 mg/L) and antipyrine (10 mg/L). The figure inside represents the kinetic model fittings at each PMS concentration. Data represent the mean values for triplicate samples with a standard deviation below 5%.

After the analysis of adsorption of antipyrine on Basolite^®^ F-300, its utility as a heterogeneous catalyst for the removal of the drug was tested in the next step of this study. Initially, the degradation rate of antipyrine (10 mg/L) was evaluated at the optimal conditions determined in the disinfection process. As is shown in Figure 4, to achieve the total degradation, more than 7 h were requested. Besides, the removal efficiency of antipyrine could be simulated as the pseudo-first-order kinetic model (Table 5). However, the antipyrine degradation rate increased when the PMS concentration was increased from 153.7 to 307.4 mg/L. A study conducted by Bi et al. [61] found the same behavior when the PMS and a Fe-MOF, particularly the MIL-101 (Fe), were used. Here, it proved that when the concentration of PMS also increases from 76.8 to 307.4 mg/L, the elimination of the pharmaceutical product, in this case, tetracycline hydrochloride, was increased [61].

**Figure 5:**
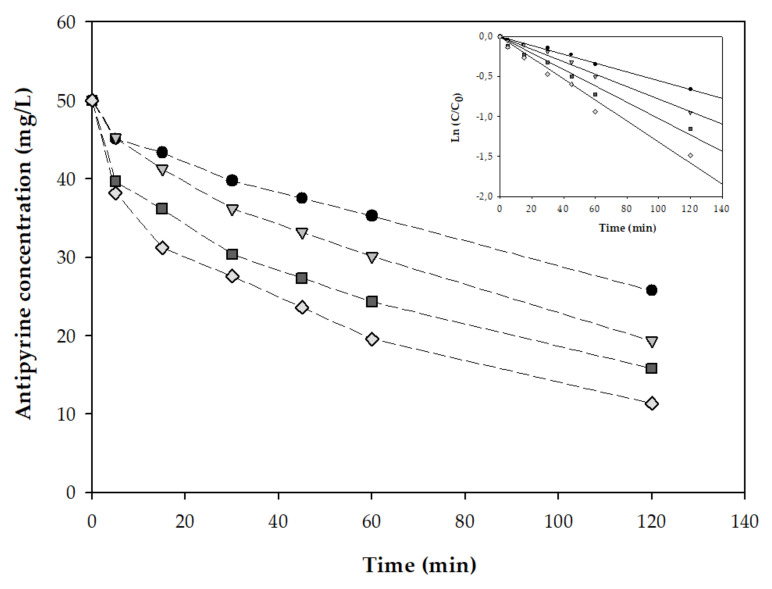
Representation of the antipyrine’s degradation profile for four different concentrations of Basolite^®^ F-300 (circle: 0 mg/L, triangle: 65.8 mg/L, square: 131.5 mg/L, and diamond: 263 mg/L). All experiments were performed with the same concentration of PMS (307.4 mg/L) and antipyrine (50 mg/L). The figure inside represents the kinetic model fittings at each Basolite^®^ F-300 concentration. Data represent the mean values for triplicate samples with a standard deviation below 5%.

The experimental results demonstrated that the PMS-Basolite^®^ F-300 system could achieve a total removal rate of antipyrine with a high-rate constant (0.013 min^−1^) within 300 min, which is 3-fold the rate obtained at 153.7 mg/L. In order to determine the effect of the Basolite^®^ F-300 concentration in the PMS activation, the degradation of a solution of 50 mg/L of antipyrine was evaluated. As mentioned in the introduction, even though the presence of these pollutants in the environment is detectable at low concentrations, several separation techniques are being utilized to concentrate these solutions, requiring the evaluation of removal techniques like the one in this study at high concentrations of pollutants. Figure 5 shows the profile of the concentration along the time using a PMS-Basolite^®^ F-300 system in a range of 307.4 mg/L PMS and Basolite^®^ F-300 from 65.8 to 263 mg/L. The observed degradation reaction rate constants of antipyrine are shown in Table 6. The antipyrine degradation rate constant significantly increased from 0.008 min^−1^ for the catalyst concentration of 65.8 mg/L to 0.013 min^−1^ for 263 mg/L. Previous studies also reported the effective degradation of MOFs by PMS activation. Similar to the obtained results in this study, Bao et al. [62] reported that the degradation rates of the MOF/PMS system increased when the dosage of catalyst increased. Besides, the TOC removal suggests that the extent of antipyrine mineralization was far lower than antipyrine degradation with a maximum mineralization level of 39.95% after 2 h with a PMS-Basolite^®^ F-300 system.

Nowadays, the number of articles trying to remove pharmaceuticals from water with PMS is still not very high. In most of them, the degradation of tetracycline is the main topic [63,64,65,66], as it is considered one of the most widely used antibiotics today. Among them, the study of Liu et al. (2022) [67] highlight the same positive effect of a catalyst (CoP/CoOx MOF) achieving practically total degradation in 15 min when a PMS concentration of 300 mg/L and 30 mg/L of a catalyst were used [67]. Similarly, the study reported by Sun et al. (2018) [68], which degrades sulphachloropyradazine with a synthesized Cobalt-based MOF, called bio-MOF-11-Co, determines the effectivity of the use of MOF as a catalyst, and the systems of MOF and PMS can degrade the drug in less than 30 min. In addition, they performed the same experiment but with other oxidizing agents, such as peroxydisulfate or peroxide, and demonstrated the high efficiency of the MOF-PMS system [68].

#### 3.2.3. *E. coli* Disinfection and Antipyrine Degradation

In the treatment of wastewater, the removal of pharmaceuticals and pathogens is necessary; without appropriate treatment, they would expose society to the danger of infection. For this reason, the efficiency of PMS-Basolite^®^ F-300 was tested in water polluted with both contaminants. Previous tests were performed to determine the effect of antipyrine on *E. coli* viability. To do that, the culture was performed following the experimental procedure describe in Section 2.2 with addition in an MPB medium of antipyrine (10 and 50 mg/L). Thus, in the control and antipyrine cultures, the CFU/mL were 2.35 × 10^10^, 2.20 × 10^10^ (10 mg/L), and 2.11 × 10^10^ (50 mg/L), respectively. These results evidenced that there was no obvious difference between the number of bacteria detected with and without antipyrine.

An *E. coli* disinfection and antipyrine degradation assay was carried using a PMS-Basolite^®^ F-300 system as described in Section 2.3.3. The PMS-Basolite^®^ F-300 system showed a high efficiency with a total removal of both pollutants after 1 h. In the literature, there are other studies such as those reported by Bai et al. [69] which have a similar idea, as they remove the same pathogen from water, *E. coli*, but in this case, the other pollutant is the dye, Rhodamine B, using a Cu MOF as the catalyst and PMS [69]. Similarly, these authors achieved the total removal of both pollutants after 1 h, which is indicative of the great activity of the PMS-MOF systems.

**Figure 6:**
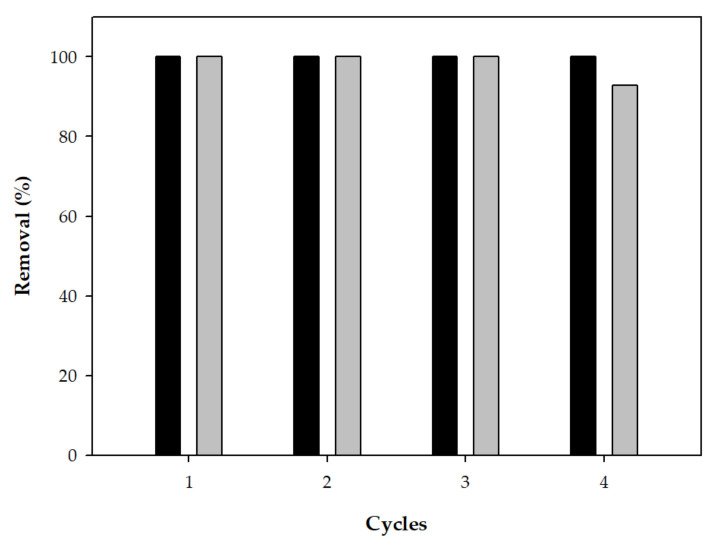
Percentage of removal of antipyrine (grey) and *E. coli* (black) obtained in four successive cycles. Data represent the mean values for triplicate samples with a standard deviation below 5%.

As the reusability of heterogeneous catalysts is an important parameter for economic and environmental considerations, the efficiency of the proposed system was performed in successive cycles. To do that, the stability and reusability of the Basolite^®^ F-300 catalyst were examined in successive cycles of the PMS-Basolite^®^ F-300 system by the addition in each cycle only of the pollutants. Concerning *E. coli*, the procedure assures a minimum concentration of 10^10^ CFU/mL in the control experiments and antipyrine was added at an initial concentration of 10 mg/L. The antipyrine and disinfection efficiency obtained for four consecutive runs is depicted in Figure 6. Similarly, in the literature, the reusability of different MOFs in several AOPs (Table 7) has been reported with no operational problems after several cycles.

**Figure 7:**
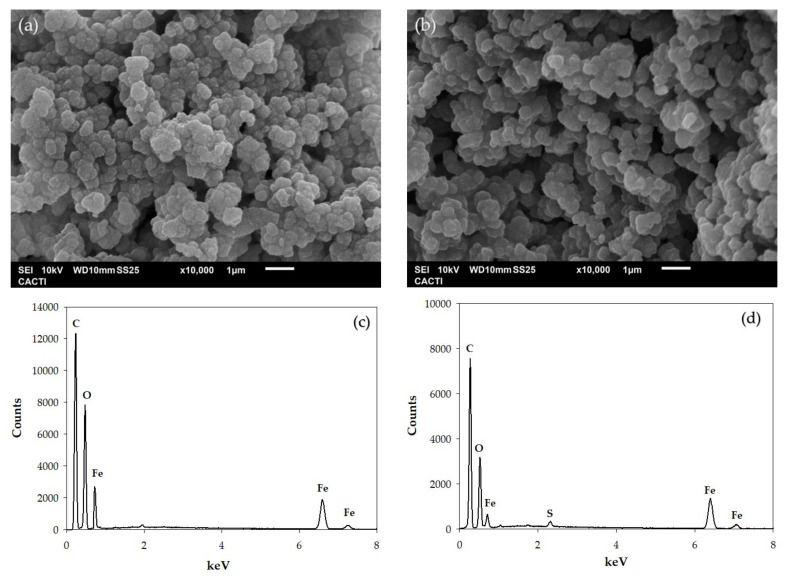
SEM-EDS images and mapping of Basolite^®^ F-300: (**a**,**c**) initial and (**b**,**d**) after four cycles.

The results showed that the disinfection efficiency was total in the four systems, and a slight decrease in the antipyrine removal (around 7.2%) observed in the last cycle could be attributed to the reduction of the generation of sulfate radicals, due to the modification of the physicochemical properties of the catalyst’s surface. Therefore, the characterization of Basolite^®^ F-300 by SEM-EDS was carried out, and the comparison between the initial and reused material is showed in Figure 7.

This figure shows the EDS mapping, demonstrating the distribution of the main components before (Figure 7c) and after (Figure 7d) four cycles. As can be seen in Figure 7a and Figure 7b, the structure remained unchanged after the material was used compared to the starting material. The EDS compositional analysis showed that the composition of Basolite^®^ F-300 varies in the main elements, which are carbon, oxygen, and iron. In this analysis, the weight percentage of carbon decreased after four cycles. Also, it was remarkable that sulfur atoms appear after these cycles (Figure 7d) because of the use of the PMS. Concerning the weight percentage of iron, a slight decrease was observed. This fact is in accordance with the low iron leaching, whose concentration in the solution was between 0.492–0.501 mg/L. Thus, the reduction in degradation efficiency is due to the need to add more PMS to maintain the degradation rate as determined in the previous experiments. Thus, the proposed system presented good performance and usability for the removal of contaminants with low leaching, and accordingly, appears to be an ideal process for the treatment of concentrated solutions with a high concentration of contaminants.

## 4. Conclusions

In summary, Basolite^®^ F-300 was tested as a heterogeneous iron catalyst to activate PMS for *E. coli* disinfection and antipyrine degradation. The effects of the amount of Basolite^®^ F-300 and PMS on *E. coli* disinfection were evaluated, and their levels optimized. The results showed that with the addition of Basolite^®^ F-300, it is possible to reduce the PMS amount achieving a high disinfection efficiency. Besides, the study of the antipyrine adsorption on Basolite^®^ F-300 showed the low ability with values lower than 7.2%, which is indicative that this MOF is not a material with suitable properties as an antypirine adsorbent. The relationship of pollutant concentrations with the ratio of PMS and Basolite^®^ F-300 is highlighted. Thus, in the treatment of concentrated solutions of antipyrine, it was necessary to increase the PMS and Basolite^®^ F-300 levels in order to increase the degradation rate. In addition, this system can be used for at least four cycles without significant loss in catalytic activity. Thus, the proposed system presented good behavior and usability for the removal of the pollutants with low leaching, and in consonance, seems to be an ideal process for the treatment of a concentrated solution with high pollutants concentration.

## Figures and Tables

**Table 1:** Factor levels of the two independent variables selected for the experimental design.

Independent Variable	Factor Level		
	−1	0	1
x_1_ (PMS) mg/L	30.7	92.2	153.7
x_2_ (Basolite^®^ F-300) mg/L	0	32.9	65.8

**Table 2:** Experimental Central Composite Experimental Design matrix and responses in actual factors. Data represent the mean values for triplicate samples with a standard deviation below 15%.

Run	PMS (mg/L), x_1_	Basolite^®^ F-300 (mg/L), x_2_	Disinfection Efficiency (5 min)
1	153.7	0.0	5.10
2	92.2	65.8	4.97
3	30.7	32.9	2.43
4	30.7	65.8	2.83
5	92.2	32.9	4.86
6	92.2	32.9	5.05
7	153.7	32.9	5.62
8	153.7	65.8	6.24
9	30.7	0.0	2.03
10	92.2	0.0	4.45
11	92.2	32.9	4.96

**Table 3:** ANOVA analysis for the response surface quadratic model in relation to disinfection efficiency.

Source	Sum of Squares	Degrees of Freedom	Mean Square	*F*-Values	*p*-Values
**Model**	18.34	4	4.58	105.45	<0.0001 significant
**x_1_**	16.03	1	16.03	368.67	<0.0001 significant
**x_2_**	0.91	1	0.91	20.86	0.0038 significant
**x_1_ x_2_**	0.003	1	0.003	0.079	0.7882
**x_1_^2^**	1.50	1	1.50	67.43	0.0011 significant
		**Std. Dev**	0.21	**R^2^**	0.986
		**Mean**	4.41	**Adj R^2^**	0.977
		**CV %**	4.73	**Pred R^2^**	0.934
			**Adeq Precision**		29.16

**Table 4:** Obtained parameters from the adsorption kinetics fittings in the removal of the antipyrine with a certain concentration of Basolite^®^ F-300.

Pseudo-First-Order Model				
[Basolite^®^ F-300] (mg/L)	[Antipyrine] (mg/L)	R^2^	*q_e_* (mg/g)	*k*_1_ (min^−1^)
65.8	10	0.989	2.27	0.383
65.8	50	0.994	6.82	0.363
263	10	0.996	4.81	0.236
263	50	0.995	12.65	0.506
**Pseudo-Second-Order Model**				
**[Basolite^®^ F-300] (** **mg/L** **)**	**[Antipyrine]** **(mg/L)**	**R^2^**	***q_e_*** **(mg/g)**	***k*** **_2_** **(g/mg·min)**
65.8	10	0.997	2.36	0.359
65.8	50	0.990	6.98	0.145
263	10	0.981	5.03	0.092
263	50	0.997	12.89	0.138

**Table 5:** First-order kinetic of antipyrine degradation; its concentration is 10 mg/L in the presence of Basolite^®^ F-300 (65.8 mg/L) and a certain concentration of PMS (153.7–307.4 mg/L).

		PMS Concentration (mg/L)	
Parameters	307.4	230.5	153.7
R^2^	0.990	0.997	0.997
*k* (min^−1^)	0.013	0.008	0.005

**Table 6:** First-order kinetics of antipyrine degradation, with a concentration of 50 mg/L in the presence of PMS (307.4 mg/L) and a certain concentration of Basolite^®^ F-300 (0–263 mg/L).

	Concentration of Basolite^®^ F-300 (mg/L)			
Parameters	263	131.5	65.8	0
R^2^	0.971	0.969	0.993	0.993
*k* (min^−1^)	0.013	0.010	0.008	0.006

**Table 7:** Summary of the results of reuse of MOF as a catalyst in various AOPs.

MOF	Pollutant	Process	Cycles/Efficiency	Ref.
Basolite^®^ F-300	Antipyrine (AP) and *E. coli*	PMS activation	AP—1st cycle: 100%, 4th cycle: 93% *E. coli*—1st cycle: 100%, 4th cycle: 100%	This study
CA@Ti-MIL-NH_2_	Paracetamol	Adsorption and degradation/ visible light	1st cycle: 96% 5th cycle: 85%	[70]
Fe_3_O_4_@MOF-525	Tetracycline (TC)	Photocatalysis	1st cycle: 98% 4th cycle: 94%	[71]
NbCo-PZ	TC	PMS activation	1st cycle: 95.5% 6th cycle: 60.4%	[72]
NH_2_-MIL-88B(Fe)@CM	Naproxen	Electro-Fenton	1st cycle: 86% 5th cycle: 86%	[73]
UiO-66-NH_2_	TC and Ketorolac tromethamine (KTC)	Photocatalysis	TC—1st cycle:71.8%, 5th cycle: 58% KTC—1st cycle: 68.3%, 5th cycle: 50%	[74]

## Data Availability

Not applicable.
